# Study on the active ingredients and mechanism of Jiaotai Pill in the treatment of primary insomnia based on network pharmacology and GEO statistics: A review

**DOI:** 10.1097/MD.0000000000035253

**Published:** 2023-09-22

**Authors:** Limin Pan, Yaolei Wang, Ruiqian Guan, Qingchun Shi

**Affiliations:** a The First Affiliated Hospital of Heilongjiang University of Traditional Chinese Medicine, Harbin, China; b Heilongjiang University of Traditional Chinese Medicine, Harbin, China; c The First Affiliated Hospital of Heilongjiang University of Traditional Chinese Medicine, Harbin, China.

**Keywords:** a comprehensive database of gene expression, Jiaotai Pill, mechanism of action, molecular docking, network pharmacology, primary insomnia

## Abstract

**Objective:**

To explore the active components and mechanism of Jiaotai Pill (JTP) in the treatment of primary insomnia (PI) based on gene expression omnibus.

**Methods:**

The main active components of Jiaotai Pills were obtained by TCMSP and literature mining, and the targets of the active components of Jiaotai Pills were predicted. The targets were verified and standardized by Uniprot database. PI-related targets were obtained from GeneCards, OMIM, DrugBank, PharmGKB, and TTD databases. Obtaining an intersection action target point of the Jiaotai pill and the PI by using a Venny diagram; Gene chip data (GSE208668) was downloaded from gene expression omnibus database, and then gene probe enrichment analysis (GSEA) was used to screen the differentially expressed genes between PI patients and normal controls, and molecular docking was used to virtually verify the screened differentially expressed genes with potential active compounds.

**Results:**

21 active components and 263 potential targets of Jiaotai Pill were screened by database analysis and literature mining, 112 of which were intersected with PI. Molecular docking results showed that quercetin, EGCG, kaempferol, R-kanatin, stigmasterol, berberine and other core active components had good docking activity with related differential genes.

**Conclusion:**

Jiaotai Pill can regulate the release of inflammatory factors through multiple active ingredients, multiple disease targets, multiple biological pathways and multiple pathways to achieve the purpose of treating PI, which provides a theoretical basis for the clinical treatment of PI and broadens the clinical use of Jiaotai Pill.

## 1. Introduction

Primary insomnia (PI) is defined as the most common sleep problem in today’s society, which excludes insomnia caused by other diseases, and is a subjective experience that patients are not satisfied with the time and quality of daily sleep, and at the same time, it has an impact on daytime work and social interaction.^[1]^ In a nationwide health plan survey of more than 10,000 members in the United States, about 30 to 40% of adults experienced insomnia symptoms, of which the prevalence of short-term insomnia was about 9.5%, and one-fifth of patients with short-term insomnia could turn into chronic insomnia.^[2]^ At the same time, the Canadian Association used the same method to study and found that the incidence of insomnia is increasing year by year. According to the analysis of national health survey data, the number of adults with insomnia symptoms increased from 13.4% in 2002 to 23.8% in 2015.^[3]^ At present, an epidemiological survey on PI in China shows that about 10% to 15% of the natural population suffer from PI.^[4]^ At the same time, insomnia is associated with a variety of diseases, including cardiovascular and cerebrovascular diseases, digestive diseases, cancer, hypertension and diabetes. Li L et al. Conducted a study on the relationship between insomnia and mental illness, in which about 40% of insomnia patients had mental illness, insomnia patients had a high risk of depression and anxiety disorders in a year, and insomnia increased the risk of suicide in depression patients.^[5,6]^ At present, the incidence of PI has increased significantly with the COVID-19 pandemic, while the risk of anxiety, depression, and suicide has increased significantly.^[7]^ For the treatment of insomnia, Western medicine recommended sedative hypnotics as the main treatment, the clinical effect is obvious, but there are dependence, addiction, liver, and kidney damage and other adverse reactions. Therefore, there are more and more traditional Chinese medicine treatments for PI. Traditional Chinese medicine treatment has exact effect, less clinical adverse reactions, no addiction, and dependence. At the same time, in the long-term medical practice, doctors of past Dynasties have provided rich clinical experience for traditional Chinese medicine treatment of PI.^[8]^

The prescription and name of Jiaotai Pill are clearly recorded in Wang Shixiong, a famous doctor in the Qing Dynasty, in the chapter of “Brief Prescriptions of Four Departments, Anshen.” “Jiaotai Pill” is used to treat insomnia due to disharmony between the heart and the kidney, which is the main prescription for the treatment of insomnia due to disharmony between the heart and the kidney, and is modified on the basis of it in clinical treatment. So far, the study of Jiaotai Pill in the treatment of PI is mainly focused on experimental animals or clinical pharmacological observation.^[9,10]^ There are many reports on the mechanism of Jiaotai Pill in the treatment of PI, and there is no clear clinical trial to verify it.^[11]^

Network pharmacology is an emerging discipline integrating systems biology, medicinal chemistry, pharmacology, and other disciplines, which can reflect the complex interaction network between “drug-gene-target-disease,” and is widely used in the interpretation of drug pairs and prescription compatibility rules.^[12]^ Molecular docking technology can obtain the affinity of small molecules and receptor proteins by computer simulation, and further realize the screening and design of drugs, which provides a reference for the clinical application of Jiaotai Pill and the subsequent research of related traditional Chinese medicine.

## 2. Materials and methods

### 2.1. Collection of main chemical components of Jiaotai Pill

The related active components of *Coptis chinensis* and *Cinnamomum cassia* in Jiaotai pills were searched by TCMSP (https://old.tcmsp-e.com/tcmsp.php) database and NCBI (https://www.ncbi.nlm.nih.gov/) database, and the related literatures were also searched. The main active ingredient selected from all active ingredients according to relevant biological function criteria.

### 2.2. Excavation and screening of active ingredients of Jiaotai Pills

Oral bioavailability (OB) and drug likeness (DL) are one of the main parameters of drug metabolism. Substances with OB ≥30% and DL index ≥0.18 are highly druggable (33). Therefore, in the TCMSP database, the chemical components with the screening conditions of OB = 30% and DL = 0.18 are selected as the candidate active components to obtain the active components of Jiaotai pills. At the same time, TCMSP and PubChem (https://pubchem.ncbi.nlm.nih.gov/) platform were used to confirm the structure of the active ingredients retrieved, and they were saved as mol2 format files.

### 2.3. Obtain the target of Jiaotai Pill and the target related to PI

The gene target information of the screened Jiaotai Pill core protein targets was mined, and the UniProt KB search function in the UniProt database (33)(https://www.uniprot.org/) was selected.^[13]^ The database selection was limited to “Reviewed (Swiss-Prot),” and the species was “Human,” and the target was queried to obtain the gene target of the active ingredient of Jiaotai Pill. The PI disease target is screened through 5 databases including Gene cards, OMIM, DrugBank, PharmGKB, and TTD, wherein the higher the relevance score value in the Gene cards database is, the closer the relationship between the target and the disease is, The target of relevance score ≥5^[14]^ was selected as the target of PI disease (36), and the reported genes related to PI were searched by inputting the keywords “Primary insomnia” or “PI,” and the duplicate and false positive genes were screened out and verified by literature search. O as to obtain a potential sedative and hypnotic mechanism target of the Jiaotai pill. The online software Venny 2. 1 mapping platform was used to draw the Venny map of PI potential target screening and Jiaotai pill active ingredient-PI potential target respectively.

### 2.4. Construction of active ingredient-PI potential target network model and PPI network of Jiaotai Pill

The information of active ingredients and targets of Jiaotai Pill was imported into Cytoscape _ v3.9.1 software to construct the interaction network diagram of “Jiaotai Pill-active ingredients-PI-targets.” The Network Analyzer of Cytoscape _ v3.9.1 software was used to calculate the constructed network graph, and the degree of the network was analyzed by statistical analysis. The larger the degree value was, the more important the node was, so as to screen out its core active component.^[15]^

The target of Jiaotai Pill was introduced into the database of functional protein association networks^[16]^ (https://cn.string-db.org/). Homo sapiens was defined as the species by using the Multiple proteins tool, and the protein interaction was obtained. In the process of obtaining, the minimum interaction threshold of protein target was set as “highest confidence” (>0.9), and the free protein was hidden and saved in TSV format. The node1 and node2 information in the file were imported into Cytoscape software to draw the protein interaction network, and the network was topologically analyzed, and the core target data was visualized to construct the PPI network diagram.

### 2.5. Gene ontology and pathway analysis

The DAVID database (https://david.ncifcrf.gov/) was used for GO analysis and Kyoto Encyclopedia of Genes and Genomes (Kyoto Encyclopedia of Genes and Genomes, KEGG) enrichment analysis of the core targets. Three modules, biological process (BP), cell component (CC), and molecular function (MF), were selected for GO enrichment analysis, and KEGG was used for pathway analysis. O as to obtain a pathway which is related to the pathogenic mechanism of the PI and enriched by most of key genes, meanwhile, the statistical significance threshold is set to be *P* < .05, the data result are subjected to visualization processing through a microbioinformation platform (http://www.bioinformatics.com.cn/), and a core target and pathway diagram is drawn, to analyze the approach of Jiaotai Pill in the treatment of PI.

### 2.6. PI sample data download

The National Center for Biotechnology Information, The gene expression omnibus (GEO) database (http://www. ncbi.nlm. nih. gov/geo) of the NCBI) searches for “Primary insomnia” samples. Inclusion criteria: samples should include PI samples and normal samples. The sample was of human tissue origin (apiens), and the chip data type was gene expression profile.^[17]^

### 2.7. Analysis and verification of differential genes

GSEA 4.1.0 was used to analyze the expression matrix annotated by GEO database for the screening of the obtained targets, and number of permutations = 1000 was set; PhenotypeLabels was PI versus normal; permutation type was phenotype, and the first 2000 was the differential gene between PI population and normal population. And take an intersection of that obtained differential gene active component and the potential target to obtain the verification target.

### 2.8. Molecular docking

The core active components screened from the network of “active components-T2DM targets” of Jiaotai Pill were selected for molecular docking with the differentially expressed intersection targets. The 3-dimensional structures of the potential targets and the screened targets to be verified are searched and obtained through the RCSB PDB database (http://www.rcsb.org/). The 3D structure of the core active compound of Jiaotai Pill was downloaded from Pubchem database (https://pubchem.ncbi.nlm.nih.gov/), and AutoDockTools 1.5.7 software was used to remove water molecules and ligands from the above file, and at the same time, hydrogenation and electron addition were carried out. The molecular docking of the core active ingredients with the potential core targets is carried out in turn.

## 3. Results

### 3.1. Screening of active ingredients in Jiaotai Pills

By setting the screening conditions of TCMSP database as OB ≥ 30% and DL ≥ 0.18, the related active components of Coptis chinensis and Cinnamomum cassia were screened, and 21 active components of Jiaotai pills were screened, including 13 components of Coptis chinensis and 8 components of Cinnamomum cassia. The main active ingredients of cinnamon are cinnamaldehyde and cinnamic acid, which are also included in the active ingredients, as shown in Table 1.

**Table 1:** Main active ingredients and some pharmacokinetic parameters in Jiaotai Pills.

TCM	Mol ID	Molecule name	OB/%	DL
Coptis Chinensis	MOL002668	Worenine	45.83	0.87
	MOL001458	coptisine	30.67	0.86
	MOL002904	Berlambine	36.68	0.82
	MOL001454	berberine	36.86	0.78
	MOL006393	epiberberine	43.09	0.77
	MOL002903	(R)-Canadine	55.37	0.77
	MOL013352	Obacunone	43.29	0.77
	MOL002894	berberrubine	35.74	0.72
	MOL000789	jatrorrhizine	30.44	0.75
	MOL000762	Palmidin A	35.36	0.65
	MOL000785	palmatine	64.60	0.64
	MOL000098	quercetin	46.43	0.28
	MOL008647	Moupinamide	86.71	0.26
Cinnamomum Cassia	MOL000004	Procyanidin B1	67.87	0.66
	MOL000449	Stigmasterol	43.83	0.76
	MOL000098	quercetin	46.43	0.28
	MOL000991	cinnamaldehyde	31.99	0.02
	MOL002295	Cinnamic acid	19.68	0.03
	MOL000359	β-sitosterol	36.91	0.75
	MOL000422	kaempferol	41.88	0.24
	MOL006821	(-)-epigallocatechin-3-gallate	55.09	0.77

### 3.2. Prediction of active ingredients of Jiaotai Pill and potential targets of PI intervention

**Figure 1:**
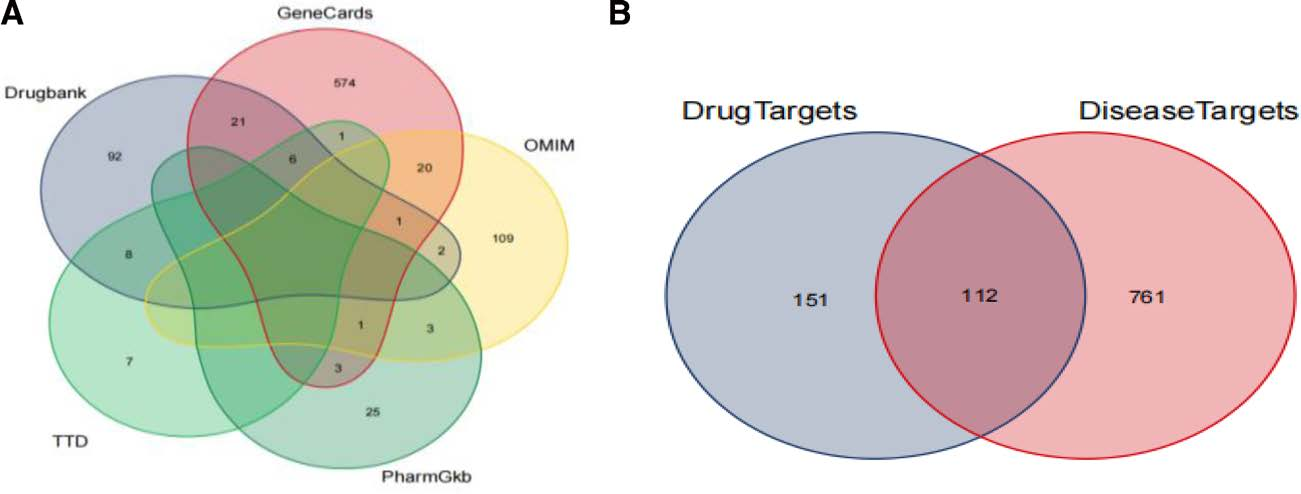
(A) Venn diagram for PI potential target screening. (B) Venn diagram for active ingredient and PI potential target of Jiaotai Pills.

The 21 core active components of Jiaotai Pill were input into the TCMSP database to obtain 300 protein targets. By inputting the name of protein target in UniProt KB function item of Uniprot database, “Reviewed (Swiss-Prot)” and “Human” were defined, and 263 gene targets were obtained. PI potential targets were predicted in GeneCards, OMIM, DrugBank, PharmGKB, TTD, and other databases, and 873 potential targets were obtained by sorting and removing duplicates. At the same time, the potential targets of screened PI and the active ingredient targets of Jiaotai Pill were mapped with the potential targets of PI by Venny 2.1 online drawing tool, and Venn Figure 1A,B was drawn, and 112 core targets were obtained.

### 3.3. Construction and analysis of active ingredient-PI action target network of Jiaotai Pill

**Figure 2:**
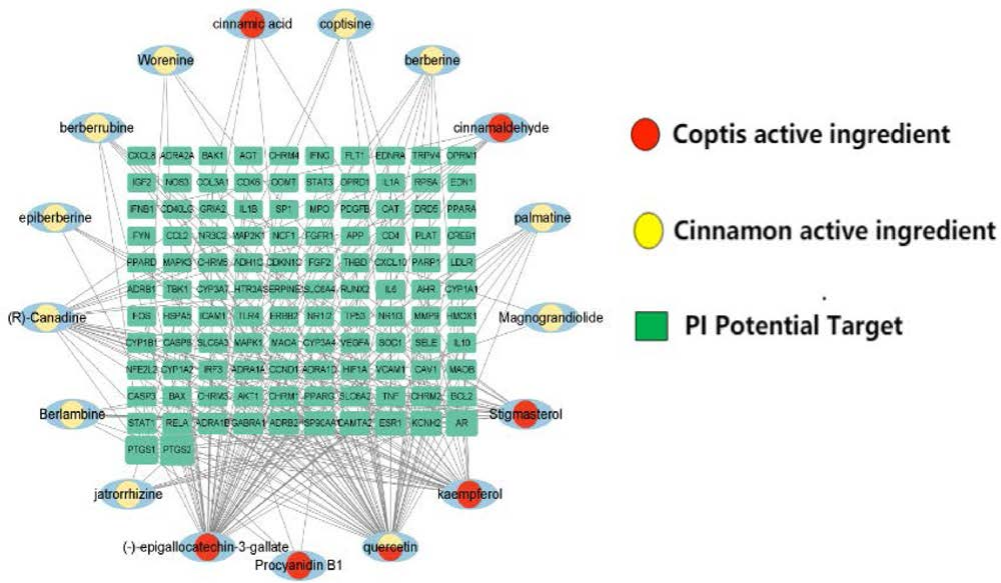
Active ingredient of Jiaotai Pills—PI potential target network.

The 21 active ingredients and 112 predicted targets of Jiaotai Pill were imported into Cytoscape _ v3.9.1 software to construct a “drug-active ingredient-PI-target” interaction network diagram (Fig. 2), including 129 nodes and 246 edges. The nodes are representatives of active ingredients and targets (green rectangular nodes represent potential targets of PI, yellow and red circular nodes represent active ingredients of Coptis chinensis and Cinnamomum cassia). The edge is the representation of the interaction relationship between the active ingredient and the target of action. A single target interacted with about 2.1 compounds, while the active ingredients interacted with an average of 3.8 targets, in which quercetin, (–) -epigallocatechin-3-gallate (EGCG), kaempferol, R-kanatin, stigmasterol, and berberine were the most corresponding targets. Interact with 61, 50, 29, 21, and 17 targets, respectively, which may be the core active component of Jiaotai Pill in the treatment of PI.

### 3.4. PPI network analysis

**Figure 3:**
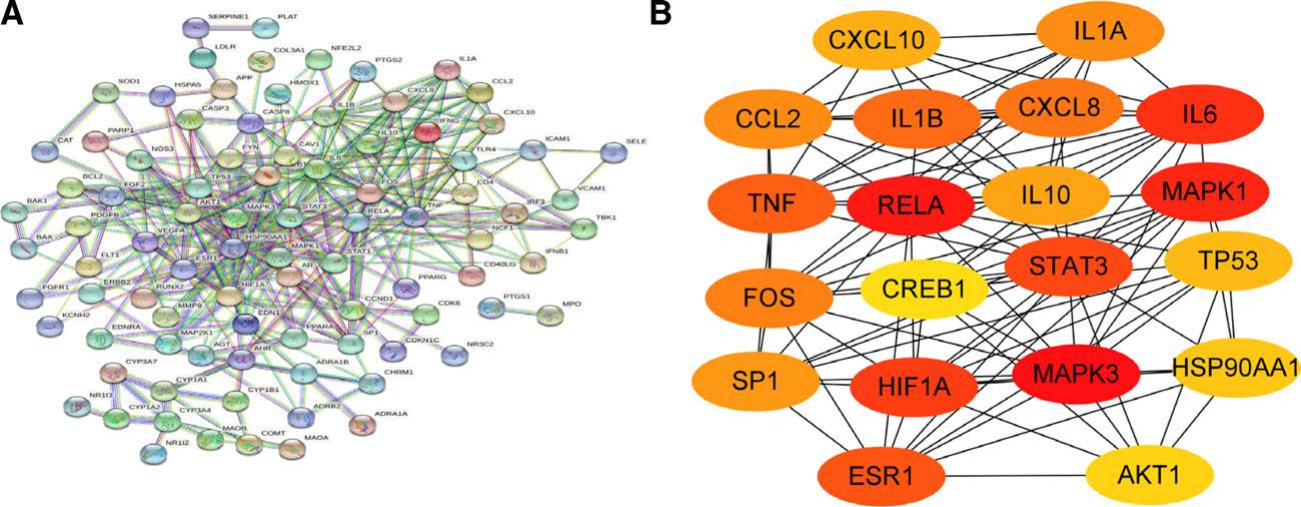
(A) Intersection gene target PPI network data. (B) Core gene target PPI network diagram.

The obtained 112 intersection targets of the active ingredients of Jiaotai Pill and PI potential targets were imported into the String database to obtain PPI network data (Fig. 3A). The network data are imported into Cytoscape _ v3.9.1 software for topological analysis, and 112 nodes and 358 edges are obtained. The MCC algorithm of Cytohubba plug-in of Cytoscape V3.9.0 software was used to process the target data, and the top 20 targets with MCC values were selected to draw the PPI network diagram (Fig. 3B). The larger the MCC value is, the closer the relationship between the drug and the target is. The color of the node represents the size of the MCC value, and the smaller the MCC value corresponding to the color changing from red to yellow. Therefore, the obtained 20 targets are taken as the core targets of Jiaotai Pill for PI treatment, as shown in Table 2.

**Table 2:** Core targets of Jiaotai Pills in the treatment of PI.

Sequence	Gene target	Core protein target	MCC value
1	MAPK3	Mitogen-activated protein kinase 3	7378
2	RELA	Transcription factor p65	7208
3	MAPK1	Mitogen-activated protein kinase 1	6926
4	IL6	Interleukin-6	6894
5	HIF1A	ypoxia-inducible factor 1-alpha	6714
6	STAT3	Signal transducer and activator of transcription 3	6690
7	ESR1	Estrogen receptor	6576
8	TNF	Tumor necrosis factor	6379
9	IL1B	Interleukin-1 beta	6126
10	CXCL8	Interleukin-8	6024
11	FOS	Protein c-Fos	5944
12	CCL2	C-C motif chemokine 2	5880
13	IL1A	Interleukin-1-alpha	5880
14	SP1	Transcription factor Sp1	5847
15	IL-10	Interleukin-10	5356
16	CXCL10	C-X-C motif chemokine 10	5070
17	TP53	ellular tumor antigen p53	1253
18	HSP90AA1	Heat shock protein HSP 90-alpha	712
19	AKT1	RAC-alpha serine/threonine-protein kinase	559
20	CREB1	Cyclic AMP-responsive element-binding protein 1	541

### 3.5. Functional analysis and pathway analysis of core target genes

**Figure 4:**
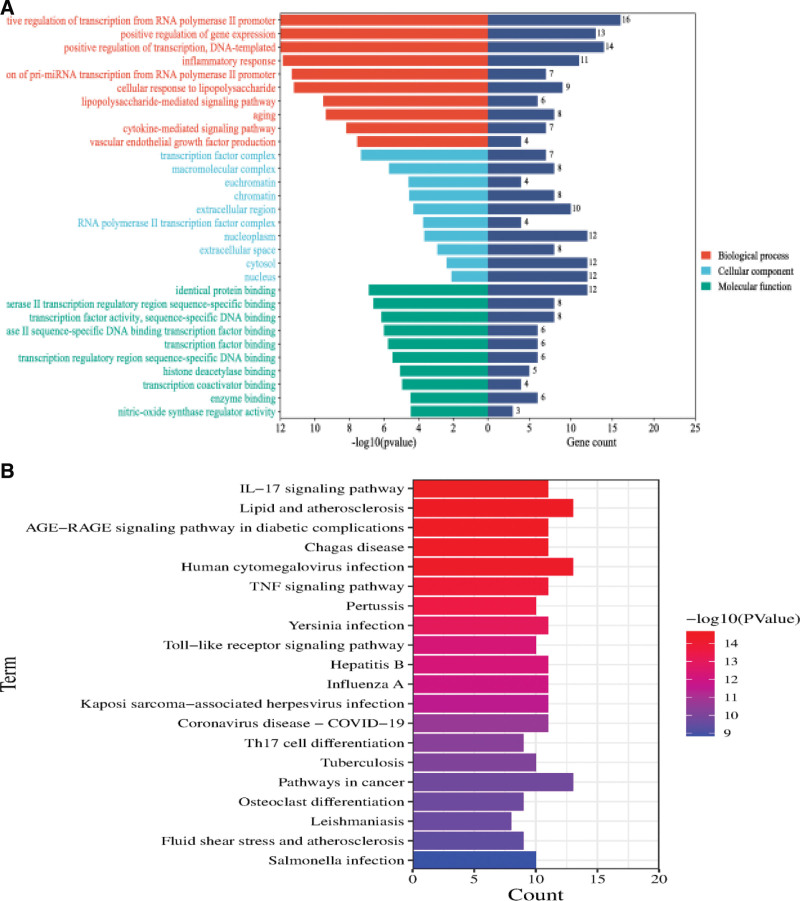
(A) GO enrichment analysis bar. (B) KEGG pathway enrichment analysis.

The key targets obtained from the PPI network were imported into the DAVID database and screened by setting (*P* < .05). The top 20 key targets ranked by MCC value were related to 247 BPs. Among them, RNA polymerase II promoter is closely related to the intuitive regulation of transcription, the positive regulation of gene expression, the positive regulation of DNA template transcription and the response to inflammation. It is closely related to 17 cellular locations (CC), including nucleus, nucleoplasm, chromatin, extracellular region, and transcription factor complex. It is related to 45 MF, among which the same protein binding, transcription factor binding, and enzyme binding are closely related. The GO functional enrichment analysis showed that Jiaotai Pill may play a role in the treatment of PI by affecting the 3 processes of BP, CC, and MF (Fig. 4A).

The key targets of KEGG pathway analysis were enriched in 139 related signaling pathways, among which the IL-17 signaling pathway was the most closely related. The second is the age−RAGE signaling pathway and TNF signaling pathway in lipid and atherosclerosis, diabetic complications, and the Toll-like receptor signaling pathway (Fig. 4B). According to the analysis results of target enrichment on the pathway-phase pathway, multiple target proteins can be enriched on 1 pathway, which fully reflects the synergistic effect of multiple targets of traditional Chinese medicine compound and plays a role of multiple pathways in disease treatment. Literature review found that IL-17 signaling pathway, TNF signaling pathway, and Toll-like receptor signaling pathway play an important role in the pathogenesis and treatment of PI.

### 3.6. Selection of differential genes and molecular docking verification

**Figure 5:**
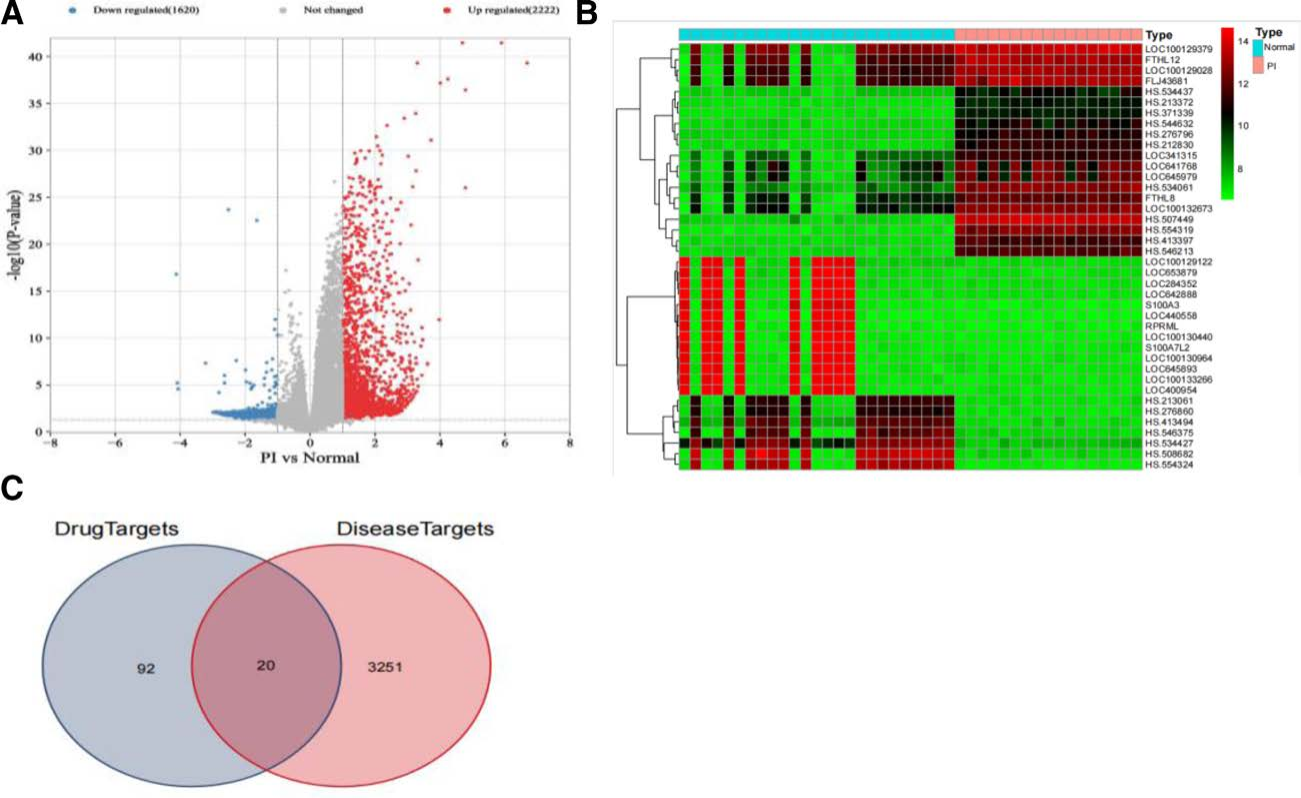
(A) Differential gene expression between PI group and normal group, volcano. (B) Heat map of differential gene expression between PI group and normal group (top 50). (C) Venn diagram of differentially expressed genes intersecting targets of PI-Jiaotai Pills.

In order to show the differential expression of genes between PI group and normal group, the volcano map of GSE208668 data set was drawn (Fig. 5A), and the heat map of the top 50 genes differentially expressed between PI group and normal group was drawn (Fig. 5B). As shown in Figure 5C, the top differential genes were intersected with the above intersection targets. Prostaglandin G/H synthase 2 (PTGS2), potassium voltage-gated channel subfamily H member 2 (KCNH2), HSP90AA1, heme oxygenase 1 (HMOX1), NF erythroid 2-related factor 2 (NFE2L2), IL-10, HIF1A, FOS, IL-6, G1/S-specific cyclin-D1 (CCND1), vascular cell adhesion protein 1 (VCAM1), cytochrome P4501B1 (CYP1B1), dwarfism-related transcription factor 2 (RUNX2), 40s ribosomal protein SA (RPSA), myeloperoxidase (MPO), interferon beta (IFNB1), CREB1, IL1B, tyrosine protein kinase Fyn (FYN), and serine/threonine protein kinase TBK1 (TBK1) were the core genes differentially expressed. Disease targets and core active ingredients were verified by molecular docking technology.

The consensus of researchers is that the binding energy of molecular docking is <0, which means that the ligand and receptor can bind spontaneously. When the binding energy is ≤–5.0 kJ/mol, the small molecule ligand and receptor bind well.^[18]^ The active ingredient with the highest correlation with the PI target in the active ingredient-PI action target network diagram of Jiaotai Pill is used as a molecular docking ligand, the core gene obtained from the PPI network and the common gene protein in the core gene screened by gene expression difference are used as a molecular docking receptor, and molecular docking is carried out by adopting AutoDockTools 1.5.7 software. The corresponding docking scores are shown in Table 3. It can be seen from Table 3 that the core active ingredients of Jiaotai Pill have the best docking activity with FOS, followed by HSP90AA1, HIF1A, CREB1, IL-6, and other targets. These targets have less binding energy with quercetin, EGCG, kaempferol, R-kanatin, stigmasterol, berberine, and other core active ingredients and the common genes screened, and the docking results are stable.

**Table 3:** Molecular docking results of PI target and core active components treated with Jiaotai Pills.

Gene target	Binding energy (/KJ mol^–1^)					
	Quercetin	EGCG	Kaempferol	R-canadine	Stigmasterol	Berberine
IL-6	–7.2	–7.1	–6.6	–7.4	–6.6	–7.8
HIF1A	–7.4	–7.4	–7.5	–8.3	–6.5	–7.3
IL1B	–6.4	–6.4	–6.3	–6.6	–6.9	–6.7
FOS	–5.4	–5.2	–6.3	–3.5	–4.5	–3.6
IL-10	–6.6	–6.5	–6.7	–7.3	–7.7	–7.4
HSP90AA1	–6.8	–7.5	–7.5	–7.4	–6.7	–7.9
CREB1	–7.2	–7.2	–6.8	–7.9	–7.1	–7.9

## 4. Discussion

In this study, the active ingredients of Jiaotai Pill were screened, the targets were predicted and the corresponding network was analyzed by network pharmacology. Combined with the gene chip data from GEO database, the core target of Jiaotai Pill in the treatment of PI was identified, and its rationality was further verified by molecular docking. Results a total of 21 active compounds were isolated from Jiaotai Pills. Jiaotai Pill has 112 potential targets for PI treatment, and 20 core targets have been screened out. Molecular docking results showed that core compounds such as quercetin, EGCG, kaempferol, R-kanatin, stigmasterol and berberine may interact with IL-6, HIF1A, IL-1B, FOS, IL-10, HSP90AA1, and CREB1 to treat PI. Jiaotai Pill has the characteristics of multicomponent, multitarget, and multipathway in the treatment of PI.

Among them, quercetin, EGCG, kaempferol, and R-kanadine corresponded to 61, 50, 29, and 21 targets, respectively. The molecular docking results showed that the docking energy of quercetin, EGCG, kaempferol, and R-kanadine with the core target was low, and the docking results were good. Modern pharmacological studies have shown that the main active components of Coptis chinensis are alkaloids, of which berberine (5%–8%) is the most abundant alkaloid, followed by R-kanatin, palmatine, coptisine and epiberberine, and jatrorrhizine^[19,20]^ is the lowest. Peng et al^[21]^ found that berberine, the main component of Coptis chinensis in Jiaotai Pill, could significantly affect the spontaneous activity time of mice and had obvious sedative and hypnotic effects. Dinesh Kumar Patel et al^[22]^found that R-kanadin can inhibit the activity of acetylcholinesterase and has antioxidant activity. The different configurations of acetylcholine in different types of neurons play a role in promoting sleep and arousal induced by exogenous stimuli.^[23]^ R-kanadine can inhibit the metabolism of acetylcholine and enhance its activity in the central nervous system, thus regulating the sleep-arousal mechanism of the body to treat insomnia symptoms. The active components of Cinnamomum cassia are also one of the hot researches in the treatment of PI in recent years. ECGC has broad prospects in the treatment of PI. EGCG can reduce the release of corticosterone to down-regulate the hypothalamic-pituitary-adrenal axis to provide anxiolytic and hypnotic effects.^[24]^ Flavonoid polyphenols quercetin and kaempferol, which belong to the same genus as ECGC, have sedative and hypnotic effects, and they can also improve sleep disorders. The intake of flavonoid polyphenols is related to the improvement of sleep quality, which can reduce anxiety behavior and increase sleep time.^[25]^

The results of this study show that Jiaotai Pill may treat PI through 112 potential targets such as MAPK3, RELA, MAPK1, IL-6, and HIF1A. PPI network analysis showed that the top 20 targets with MCC value in the PPI network formed by 112 potential targets were considered as the core targets of Jiaotai Pill in the treatment of PI. The GO function enrichment analysis of the screened core targets showed that they were involved in multiple BPs, cell composition, and MFs, such as the intuitive regulation of RNA polymerase II promoter on transcription, the positive regulation of gene expression, the positive regulation of DNA template transcription, inflammatory response, nucleus, chromatin, extracellular region, same protein binding, enzyme binding, and so on. At the same time, the KEGG pathway enrichment analysis of core targets was carried out, and the potential core targets were enriched in 139 pathways, and the most enriched targets were IL-17 signaling pathway. IL-17 signaling pathway produces IL-17, a highly versatile proinflammatory cytokine that is critical for host defense, the pathogenesis of inflammatory diseases, and the progression of cancer. Enhanced activation of the IL-17 signaling pathway after sleep deprivation promotes the production of proinflammatory cytokines, leading to a series of inflammatory responses in the body.^[26]^ Studies^[27]^ have shown that TNF, a mediator of TNF signaling pathway, has a role in sleep regulation and brain development, while Toll-like receptor signaling pathway is closely related to PI. Animal experiments have shown that Toll-like receptors activate their downstream pathways and signal to activate the nuclear factor [NF]-κB signaling pathway in sleep deprivation models, and that TNF-α/NF-κB signaling pathway activation is enhanced in sleep deprived rats, both of which are responsible for neuroinflammation and are involved in the pathogenesis of sleep.^[28]^ At the same time, the inhibition of IL-17/TNF-α/Toll-like receptor signaling pathway can also inhibit the production of inflammatory factors,^[29]^ suggesting that the inhibition of IL-17/TNF-α/Toll-like receptor signaling pathway can stabilize the sleep-wake mechanism and reduce the release of inflammatory factors. Inhibiting the hyperactivation of hypothalamus-pituitary-adrenal axis may be the mechanism of Jiaotai Pill in the treatment of PI.

“Drug-active ingredient-PI-target” network results showed that 21 effective active ingredients were screened in Jiaotai Pill, which mainly used quercetin, EGCG, kaempferol, R-kanatin, stigmasterol, berberine, and other core effective active ingredients to combine with 20 potential core targets to treat PI. The potential core targets screened in the PPI network are intersected with the targets obtained in the GEO database, and are subjected to molecular docking verification with the core active component rows, and the result shows that all active components can be spontaneously combined with the intersected targets, The binding energies of quercetin, R-kanatin, kaempferol with HIF1A, EGCG with HSP90AA1, stigmasterol with IL-10, and berberine with HSP90AA1 and CREB1 were low, and there were multiple hydrogen bonds.

To sum up, Jiaotai Pill may act on 20 core targets such as IL-6, HIF1A and IL1B through 21 effective active ingredients such as quercetin, EGCG, and kaempferol in Coptis chinensis and Cinnamomum cassia, and treat PI by affecting a variety of BPs, cell composition, and MFs in conjunction with corresponding signaling pathways. Its specific mechanism of action is to combine active ingredients such as quercetin, EGCG, kaempferol, R-kanatin, stigmasterol, and berberine with core targets such as MAPK3, IL-6, HIF1A, and TNF, inhibit IL-17/TNF-α/Toll-like receptor signaling pathway and enhance the body’s sedation threshold. Inhibit the activation of the hypothalamic-pituitary-adrenal axis. In this study, network pharmacology was used as a research method to systematically analyze and summarize the pharmacological effects of the active ingredients of Jiaotai Pills on regulating sleep-wake mechanism through their corresponding target proteins, and the GEO database was used to screen the differentially expressed genes between PI patients and ordinary people, and to intersect with the drug target genes. The rationality of this study was verified by molecular docking technology. This study is of great value to the clinical application of Jiaotai Pill.

## Author contributions

**Conceptualization:** Limin Pan.

**Data curation:** Limin Pan, Yaolei Wang, Ruiqian Guan, Qingchun Shi.

**Formal analysis:** Limin Pan.

**Funding acquisition:** Limin Pan, Yaolei Wang, Qingchun Shi.

**Investigation:** Limin Pan, Qingchun Shi.

**Methodology:** Limin Pan.

**Project administration:** Limin Pan.

**Resources:** Limin Pan, Qingchun Shi.

**Software:** Limin Pan, Qingchun Shi.

**Supervision:** Limin Pan, Qingchun Shi.

**Validation:** Limin Pan, Qingchun Shi.

**Visualization:** Limin Pan.

**Writing—original draft:** Limin Pan, Yaolei Wang, Ruiqian Guan, Qingchun Shi.

**Writing—review and editing:** Limin Pan, Qingchun Shi.
